# High CD4‐to‐CD8 ratio identifies an at‐risk population susceptible to lethal COVID‐19

**DOI:** 10.1111/sji.13125

**Published:** 2021-12-13

**Authors:** Marco De Zuani, Petra Lazničková, Veronika Tomašková, Martina Dvončová, Giancarlo Forte, Gorazd Bernard Stokin, Vladimir Šrámek, Martin Helán, Jan Frič

**Affiliations:** ^1^ International Clinical Research Center St. Anne's University Hospital Brno Brno Czech Republic; ^2^ Department of Biology Faculty of Medicine Masaryk University Brno Czech Republic; ^3^ Department of Anaesthesiology and Intensive Care Faculty of Medicine Masaryk University Brno Czech Republic; ^4^ Celica BIOMEDICAL Ljubljana Slovenia; ^5^ Institute of Hematology and Blood Transfusion Prague Czech Republic

**Keywords:** CD4‐to‐CD8 ratio, COVID‐19, Intensive care unit, SARS‐CoV‐2, T cells, TEMRA

## Abstract

Around half of people with severe COVID‐19 requiring intensive care unit (ICU) treatment will survive, but it is unclear how the immune response to SARS‐CoV‐2 differs between ICU patients that recover and those that do not. We conducted whole‐blood immunophenotyping of COVID‐19 patients upon admission to ICU and during their treatment and uncovered marked differences in their circulating immune cell subsets. At admission, patients who later succumbed to COVID‐19 had significantly lower frequencies of all memory CD8+ T cell subsets, resulting in increased CD4‐to‐CD8 T cell and neutrophil‐to‐CD8 T cell ratios. ROC and Kaplan‐Meier analyses demonstrated that both CD4‐to‐CD8 and neutrophil‐to‐CD8 ratios at admission were strong predictors of in‐ICU mortality. Therefore, we propose the use of the CD4‐to‐CD8 T cell ratio as a marker for the early identification of those individuals likely to require enhanced monitoring and/or pro‐active intervention in ICU.

## 1 INTRODUCTION

The COVID‐19 pandemic, caused by SARS‐CoV‐2 infection, has proven extremely challenging to manage, both epidemiologically and therapeutically. Individual patients respond highly heterogeneously to infection, with most remaining asymptomatic or showing only mild symptoms. Of those who do develop symptomatic SARS‐CoV‐2 infection, approximately 3% require hospitalization, with one in five of these going on to require artificial ventilation and admission to the intensive care unit (ICU); unfortunately, around 35% of these most seriously ill patients will die in ICU.^1^, ^2^ At present, we do not fully understand the immunological basis of individuals’ susceptibility to SARS‐CoV‐2. In the long term, we need to be able to identify healthy individuals in the population at most risk of severe COVID‐19 if they were to become infected, in order to protect them.^3^, ^4^ However, in the short term, there is an urgent clinical need for a screening approach that can prospectively identify those already‐sick patients at greatest risk of progressive worsening and eventual death from COVID‐19 to prioritize them for intensive monitoring and more aggressive/pro‐active treatments at an earlier stage.

Various studies have described dysregulation of circulating immune cell populations in COVID‐19 patients. However, to date the majority have analysed heterogeneous cohorts with very low mortality and focused on purified peripheral blood mononuclear cells (PBMCs), thereby excluding potentially important granulocytic populations. As a consequence, we lack an integrated picture of the immune cell populations in the peripheral blood of the sickest COVID‐19 patients.^5^, ^6^ Those studies of the PBMCs from severe COVID‐19 patients showed a marked lymphopaenia associated with a strong activation and exhaustion of T cells, possibly caused by the onset of an inflammatory cytokine storm.^5^, ^7^ Studies on the monocytic compartment of severe COVID‐19 cases found that circulating monocytes are characterized by impaired functionality, showing altered cytokine release and reduced expression of HLA‐DR and CD40 often correlating with disease severity.^8^, ^9^, ^10^, ^11^ Alongside, clinical studies on severe COVID‐19 patients indicate that low CD8+ T cell counts and high neutrophil‐to‐lymphocyte ratios are associated with disease severity and mortality.^12^, ^13^, ^14^

To fill the current knowledge gap and provide a comprehensive overview of the peripheral blood immune compartment of critically ill COVID‐19 patients, we performed whole‐blood T cell and myeloid cell immunophenotyping of 19 individuals at the point of their admission to ICU. For those that survived, we monitored their blood for the changes that accompanied their recovery, through the successful weaning from the mechanical ventilation and upon eventual release from the ICU. We used these data to ask how blood immune cell populations changed during the recovery of survivors, and whether there were differences between patients at admission that were associated with either subsequent recovery or death from COVID‐19.

## 2 MATERIALS AND METHODS

### 2.1 Cohort design

In this prospective, observational cohort study, adult patients with respiratory failure due to proven COVID‐19 ARDS who were admitted to the intensive care unit (ICU) at St. Anne's University Hospital in Brno, Czech Republic from March to October 2020 were consecutively enrolled. Patients with chronic immunosuppression were excluded. All patients were treated with ‘lege artis’ supportive therapy according to current guidelines. Of the drugs with a presumed effect on COVID‐19, only dexamethasone and remdesivir were administered in the indicated cases. No other experimental therapy was used.

### 2.2 Sample collection and preparation

As at the beginning of this pandemic, little information was available on the biosafety of blood samples obtained from severe SARS‐CoV‐2‐infected individuals, we decided to inactivate the samples with 0.5% PFA directly at the ICU premises. Blood samples were collected in BD Vacutainer^®^ Tubes containing sodium heparin, and 0.5% PFA was directly added to each sample. Blood was then processed in a BSL‐2 cabinet with Cal‐Lyse lysing solution (Invitrogen) following manufacturer's recommendations. Samples were finally resuspended in FBS +10% DMSO in the original blood volume and bio‐banked at −80°C.

### 2.3 Flow cytometry

Frozen samples were allowed to thaw on ice. 100 µL of the original whole‐blood volume were washed twice with FACS buffer (PBS + 0.2% FBS, 2 nmol/L EDTA) and incubated in the dark for 30’ on ice in FACS buffer with the antibody cocktails indicated in Table S1. After the incubation with the antibodies, samples were washed with FACS buffer and centrifuged for 10’ at 300 *g* 20 µL (20 600 beads) of Precision Count Beads (Biolegend) were added to each sample immediately before the acquisition.

Samples were acquired on a Sony SA3800 spectral analyzer (Sony Biotechnologies). Data were analysed with FlowJo v10.7.1 (BD Life Sciences).

### 2.4 Statistical analyses

All the analyses were performed with Prism v8.1 (GraphPad Software). Multiple comparisons between different time points were performed with a Kruskal‐Wallis test followed by Dunn's correction. Comparisons between survivors and non‐survivors were performed with the non‐parametric Mann‐Whitney rank test. Survival curves were compared using a Log‐rank Mantel‐Cox test. Correlation analyses were performed with a non‐parametric Spearman rank correlation or with a Pearson correlation when evaluating categorical variables. *P*‐values < .05 were considered significant. Asterisks in the figures indicate the level of significance: *P ≤ .05, **P ≤ .005, ***P <.001.

## 3 RESULTS

### 3.1 Cohort characteristics

For this study, we enrolled 19 COVID‐19 patients at the time of their admission to the ICU of the St. Anne's University Hospital in Brno, Czech Republic. The cohort was strongly male‐biased (94.7% males) with an average age of 67 years, and a global in‐ICU mortality of 52.6% (Table 1). At admission, all patients were mechanically ventilated due to moderate to severe acute respiratory distress syndrome (ARDS) according to Berlin definition (average PaO_2_/FiO_2_ = 123.4), and 5 of them had the consequent need for extracorporeal membrane oxygenation (ECMO) therapy.

**TABLE 1:** Cohort characteristics

		Total	Survivors	Deceased
COVID‐19 ICU patients	n	19 (100%)	10 (52.6%)	9 (47.4%)
Sex	Female	1 (5.3%)	0 (0%)	1 (11.1%)
	Male	18 (94.7%)	10 (100%)	8 (88.9%)
Age, mean (range)		68 (47‐78)	67.6 (47‐77)	66 (51‐78)
Comorbidities, mean (range)		2.3 (0‐5)	1.9 (0‐4)	2.8 (1‐5)
	Hypertension	15 (78.9%)	9 (90%)	6 (66.7%)
	Diabetes	7 (36.8%)	2 (28.6%)	5 (71.4%)
	Obesity (BMI >30.1 kg/m^2^)	11 (57.9%)	5 (50%)	6 (66.7%)
	Chronic lung disease	4 (21%)	1 (10%)	3 (33.3%)
	Chronic ischaemic limb artery disease	1 (5.3%)	1 (10%)	0
	Ischaemic heart disease ‐ heart failure	2 (10.5%)	2 (20%)	0
	Cancer	3 (15.8%)	2 (20%)	1 (11.1%)
BMI (kg/m^2^), mean (range)		32.3 (25.4‐46.1)	31.1 (26.1‐37)	33.1(25.4‐46.1)
SOFA, mean (range)		9.79 (3‐16)	9 (5‐12)	10.7 (3‐16)
CRP (mg/L), mean (range)		192.6 (37.8‐344.9)	202.6 (79.1‐344.9)	181.4 (37.8‐341.7)
Creatinine (μmol/L) (range)		118.1 (54‐213)	101.9 (61‐194)	136.3 (54‐213)
Leucocyte count (10^9^/L), mean		19.97	27.75	12.21
Horowitz index (PaO_2_/FiO_2_), mean		123.4	122.2	124.8
ECMO	Days on ECMO, mean (range)	5 (26.3%)	2 (20%)	3 (33.3%)
		9.6 (7‐15)	7.5 (7‐8)	11 (7‐15)
Bacterial superinfection	n	10 (52.6%)	6 (60%)	4 (44.4%)
Fungal infection	n	5 (26.3%)	3 (30%)	2 (22.2%)
Antibiotic therapy	n	17 (89.5%)	9 (90%)	9 (88.8%)
Remdesivir		7 (36.8%)	5 (50%)	2 (22.2%)
Dexamethasone		8 (42.1%)	5 (50%)	3 (33.3%)

Note

Parameters are referred to the time of ICU admission.

Abbreviations: BMI, body mass index; CRP, C‐reactive protein; ECMO, extracorporeal membrane oxygenation; SOFA, sequential organ failure assessment score.

We collected blood samples within 24 hours of ICU admission (n = 19) and, for those who survived (n = 10), at the time of complete resumption of spontaneous ventilation (n = 8 samples acquired), and upon their release from ICU (n = 4 samples acquired). We then performed whole‐blood immunophenotyping using flow cytometry labelling panels designed to analyse T cell and myeloid cell subsets (Table. S1). The gating strategies used to identify each subset are presented in Figure S1, Figure S2 and Figure S3.

### 3.2 T cell subsets differ significantly between COVID‐19 subsequent survivors and non‐survivors at point of ICU admission

**FIGURE 1:**
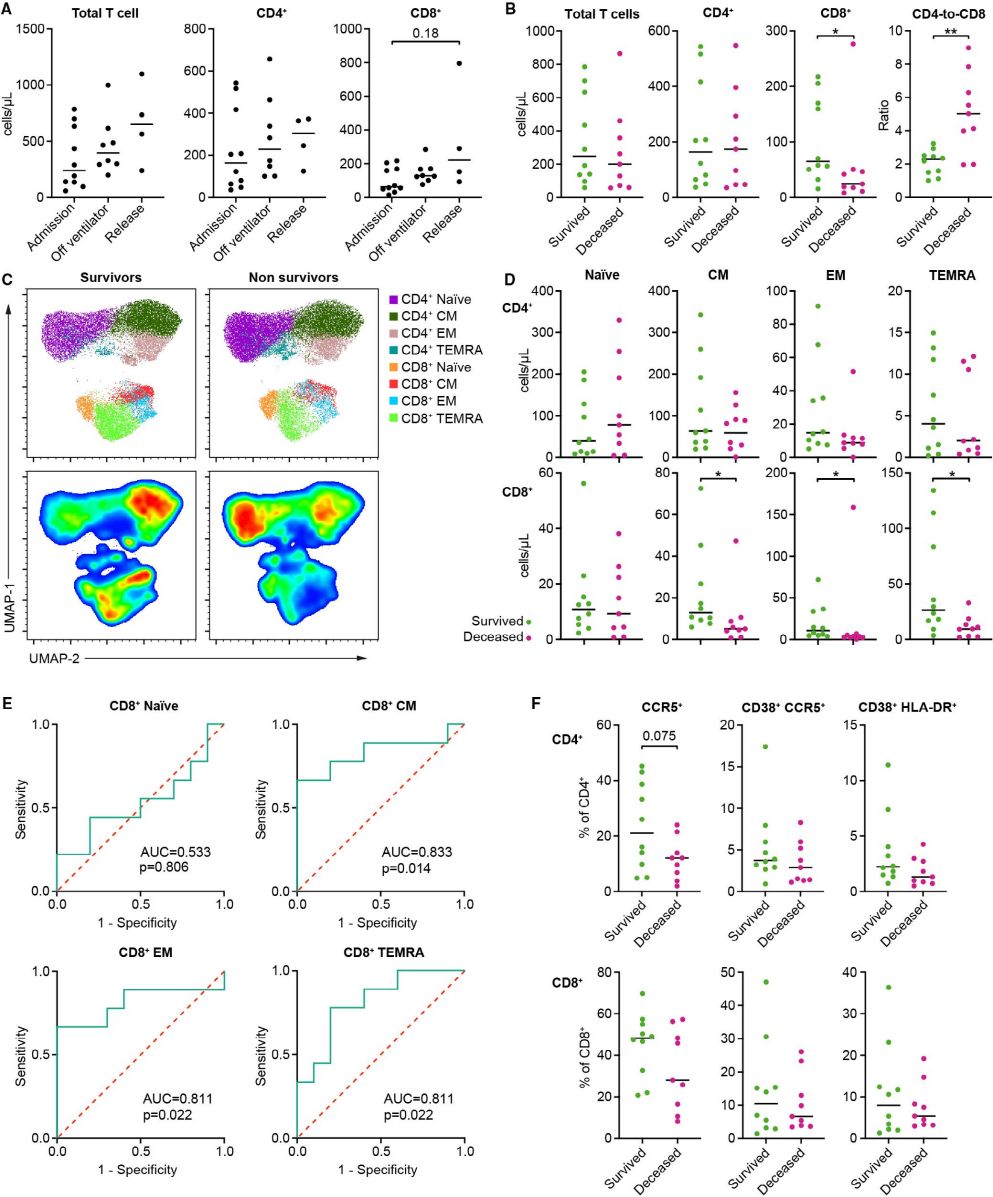
T cell subset alterations in COVID‐19 survivors and non‐survivors. A, Cell counts of total T cells, and CD4+ and CD8+ T cell subsets at different time points in blood from severe COVID‐19 survivors (n = 10, 8 and 4 at admission, restoration of spontaneous ventilation and release from ICU respectively). B, Cell counts of total T cells, CD4+ and CD8+ T cell subsets, and CD4‐to‐CD8 ratio comparison at ICU admission between severe COVID‐19 survivors and non‐survivors. C, UMAP clustering of the CD3+ population in survivors and non‐survivors. D, Comparison of naïve (CD27+ CD45R+), CM (CD27+ CD45RA‐), EM (CD27‐ CD45RA‐) and TEMRA (CD27‐ CD45RA+) subset cell counts among CD4+ and CD8+ T cells, measured at ICU admission of subsequent survivors and non‐survivors. E, ROC curves for the total counts of main CD8+ T cell subsets. F, Frequency of CD38+ HLA‐DR+, CD38+ CCR5+ and CCR5+ T cells among CD4+ (top) and CD8+ (bottom) T cells. Data in B, D and F were analysed by Mann‐Whitney test. Asterisks indicate the level of significance. *P < .05, **P < .01

Firstly, we observed that total T cell counts and proportions of CD4+ and CD8+ T cells among total T cells did not change during the initial stages of in‐ICU recovery of COVID‐19 survivors (Figure 1A, Figure S3A). However, we saw that non‐survivors had a significantly higher ratio of CD4‐to‐CD8 T cells compared with survivors at the point of admission to ICU, which was mostly due to a decreased number of circulating CD8+ T cells in non‐survivors (Figure 1B, Figure S3A). As a corroboration of previous studies,^13^ receiver operator curve (ROC) analysis showed that low CD8+ T cell count is a predictor of in‐ICU mortality of severe COVID‐19 cases (Figure S3B).

Next, we compared the subset composition of the CD4+ and CD8+ T cell populations in survivors and non‐survivors at the time of ICU admission. Visual inspection of UMAP clustering indicated that several subsets of CD4+ and CD8+ T cells differed in abundance between survivors and non‐survivors (Figure 1C). Closer analysis of the T cell subsets showed significantly lower numbers of CD8+ central memory (CM, CD27+ CD45RA‐), effector memory (EM, CD27‐ CD45RA‐) and terminal effector memory T cells re‐expressing CD45RA (TEMRA, CD27‐ CD45RA+), but not naïve T cells (CD27+ CD45RA+), in non‐survivors (Figure 1D, Figure S4C). Interestingly, the fraction of CD8+ TEMRA‐expressing CD57 was comparable between the two groups, indicating a similar degree of TEMRA exhaustion (Figure S4D). Finally, ROC analyses demonstrated that CD8+ CM, EM and TEMRA counts were good predictors of in‐ICU death (Figure 1E).

To rule out that the observed decrease in CD8+ T cells could be due to an increased migration of such cells to the inflamed sites, we measured the level of CCR5+ T cells in our cohort. We did not observe any significant difference in CCR5+ T cells nor activated CD38+ CCR5+ T cells at the time of ICU admission between patients who survived and those who did not (Figure 1F, Figure S4E). Similarly, we did not observe any difference in the frequency of activated CD38+ HLA‐DR+cells in both CD4+ and CD8+ T cell subsets (Figure 1F, Figure S4E).

Taken together, these results indicate that patients who do not survive severe COVID‐19 display T cell subset imbalances at the onset of the critical phase of their disease, particularly showing decreased CD8+ frequency which is possibly due to lowered counts of CD8+ EM, CM and TEMRA. This is consistent with a recent study showing that CD8+ T cell counts negatively correlate with disease severity,^15^ although lacking the comparison between survivors and non‐survivors presented here.

### 3.3 Monocyte frequencies and HLA‐DR expression increase with COVID‐19 recovery

**FIGURE 2:**
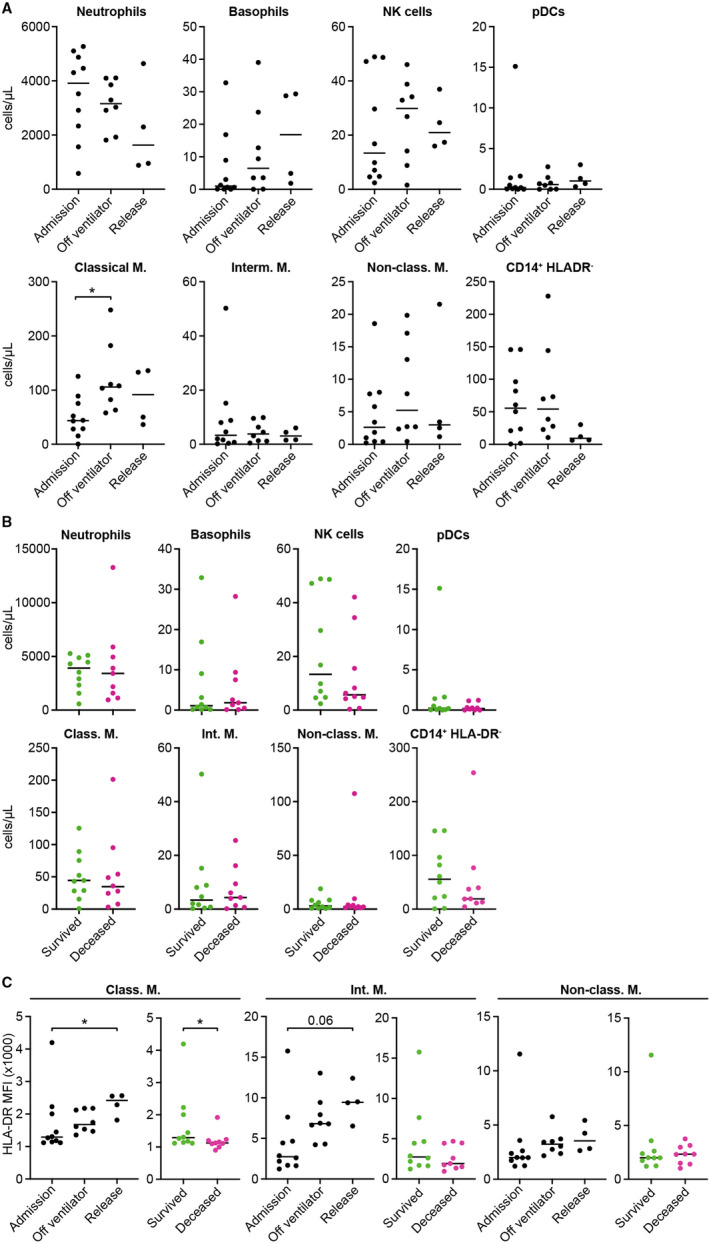
Differences in myeloid subsets between COVID‐19 survivors and non‐survivors. A, Total cell counts for granulocytes, NK cells, plasmacytoid DCs (pDCs) and classical (Classical M.), intermediate (Interm. M), non‐classical (Non‐class. M.) and CD14+ HLADR monocytes in severe COVID‐19 survivors at different time points. B, Comparison of granulocyte and other myeloid cell subset counts at ICU admission between severe COVID‐19 survivors and non‐survivors. C, Changes in HLA‐DR mean fluorescence intensity (MFI) at different time points (left) and at ICU admission between severe COVID‐19 survivors and non‐survivors (right) in each monocyte subset. Multiple comparisons (panel A, C) were computed by Kruskal‐Wallis test with Dunn's correction. Single comparisons (panel B, C) were computed by Mann‐Whitney test. Asterisks indicate the level of significance. *P < .05

Among the different myeloid cell subsets, we observed that the concentration of classical monocytes in the blood of patients who survived was low upon admission to ICU but significantly increased at the time of restoration of spontaneous ventilation (Figure 2A). However, we did not detect any differences in granulocyte (neutrophils and basophils), NK cell or pDC concentration during in‐ICU recovery stages of survivors. Similarly, we found that non‐lymphocyte cell concentrations were comparable between survivors and non‐survivors at ICU admission (Figure 2B). When we examined the monocyte subsets of survivors more closely, we observed significant changes in HLA‐DR levels in the classical and intermediate monocyte fraction, being reduced during the acute phase of the pathology (ICU admission) but increasing at later time points (Figure 2C). Similarly, we detected lower HLA‐DR levels on classical monocytes in non‐survivors (Figure 2C). Although we did not observe any difference in the levels of CD14^+^ HLA‐DR^‐^ immunosuppressive monocytes (Figure 2B), our results concerning survivors are in accordance with recent studies demonstrating that severe COVID‐19 cases showed profound dysregulation of the circulating monocyte subsets, leading to the downregulation of HLA‐DR and, consequently, the accumulation of dysfunctional CD14^+^ HLA‐DR^lo^ monocytes.^9^, ^11^, ^16^

### 3.4 CD4‐to‐CD8 T cell ratio and neutrophil‐to‐CD8 T cell ratio predict in‐ICU mortality

**FIGURE 3:**
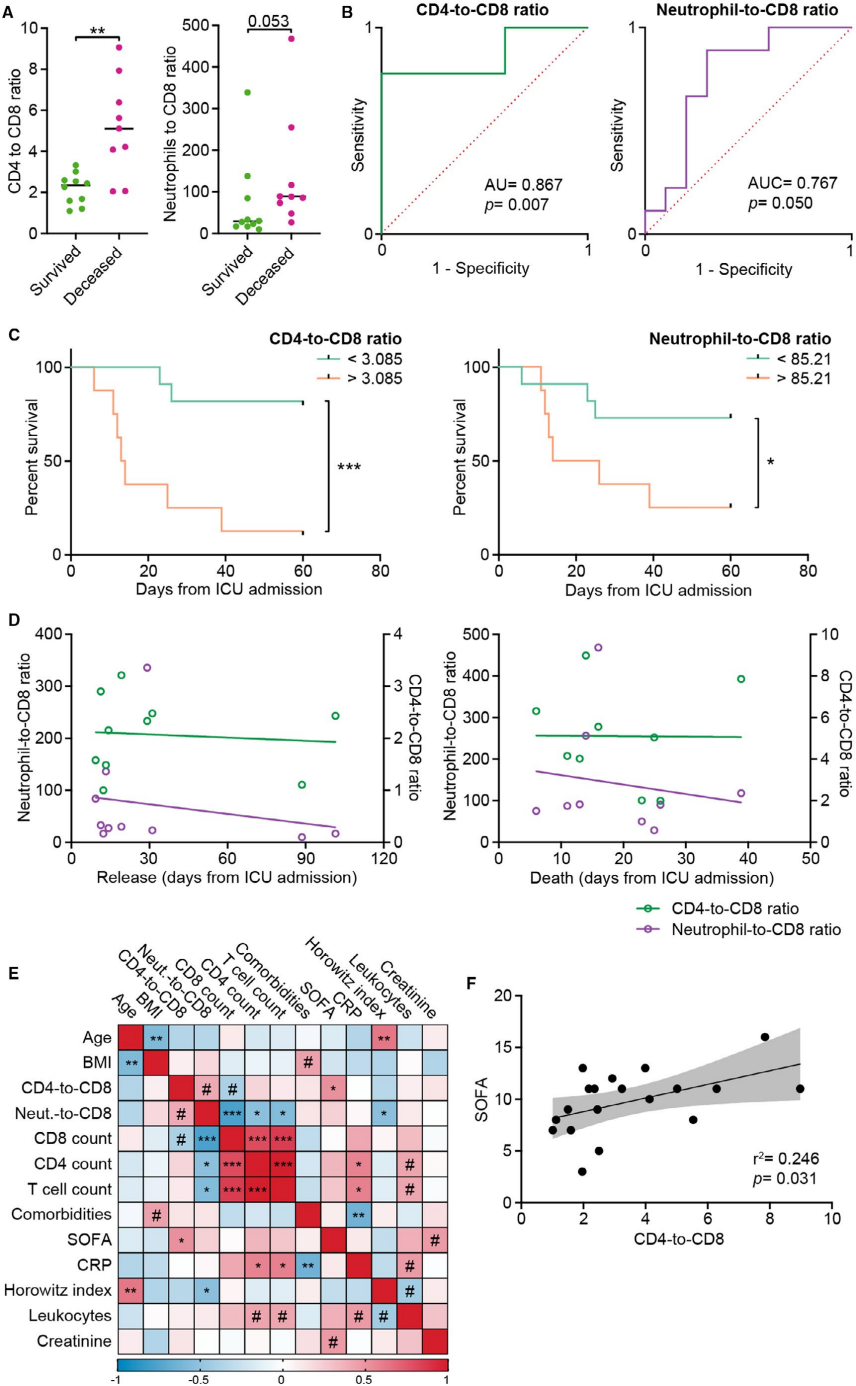
CD4‐to‐CD8 and neutrophil‐to‐CD8 ratios predict ICU mortality of severe COVID‐19 patients. A, CD4‐to‐CD8 ratio and neutrophil‐to‐CD8 ratio measured at ICU admission in COVID‐19 survivors and non‐survivors. B, ROC curves for CD4‐to‐CD8 ratio and neutrophil‐to‐CD8 ratio. C, Kaplan‐Meier analysis of CD4‐to‐CD8 ratio and neutrophil‐to‐CD8 ratio measured at the time of ICU admission. Thresholds were set to match the highest likelihood ratio. D, Correlation between CD4‐to‐CD8 ratio (green) or neutrophil‐to‐CD8 ratio (purple) and length of stay in ICU for survivors (left panel) and non‐survivors (right panel). Lines were drawn using linear regression. E, Correlation matrix between different measured parameters. The colour of each box indicates the Spearman r values. Symbols indicate the level of significance. ^#^ *P* < .1, *P < .05, **P < .01, ***P < .001. F, Plot of the Spearman correlation between the SOFA score and the CD4‐to‐CD8 ratio. The line was drawn using linear regression. Grey area indicates 95% confidence interval of the best fit line. *P* value indicates the *F* test result testing the null hypothesis of a zero‐slope line. Data in a were analysed by Mann‐Whitney test. Survival curves in c were compared with a Log‐rank Mantel‐Cox test. Correlations in D, E and F were calculated by a Spearman correlation test. Asterisks indicate the level of significance. *P < .05, **P < .01, ***P < .001

As CD8+ T cells were the most affected subset in our COVID‐19 cohort, we asked whether the CD4‐to‐CD8 T cell ratio or the neutrophil‐to‐CD8 T cell ratio could be used as a prognostic marker for COVID‐19 in‐ICU mortality. We found that both ratios were higher in non‐survivors compared with survivors at the point of ICU admission (Figure 3A): the area under the curve (AUC) of ROCs was 0.867 (*P* = .007) and 0.767 (*P* = .050) respectively (Figure 3B). Furthermore, Kaplan‐Meier analyses demonstrated that both the parameters were good predictors of in‐ICU mortality for severe COVID‐19 patients (Figure 3C). In healthy individuals, the CD4‐to‐CD8 ratio ranges between 1.5 and 2.5 and is known to increase with age.^17^, ^18^ Although a low or inverted ratio is considered an immune risk phenotype,^19^ here, we show that patients with a CD4‐to‐CD8 ratio higher than 3 were more likely to succumb to severe COVID‐19 (Figure 3C). Consistent with other studies showing that higher neutrophil‐to‐CD8 ratios were predictive of a severe COVID‐19 course,^20^ we found that a neutrophil‐to‐CD8 ratio higher than 85.2 was also predictive of in‐ICU mortality (Figure 3C). Although neither of the ratios correlated with a longer stay in ICU (for survivors) nor with the time from ICU admission to death (Figure 3D), we found that the CD4‐to‐CD8 ratio positively correlated with the sequential organ failure assessment (SOFA) score (Figure 3E,F). Similarly, we observed that the CD4‐to‐CD8 ratio was the only parameter correlating with in‐ICU mortality (Table S2). Although the CD4‐to‐CD8 ratio is known to rise with age,^18^ we did not observe any correlation between the CD4‐to‐CD8 ratio and the age of our patients, or any correlation between age and mortality (Figure 3E, Figure S5A, Table S2). Finally, while the neutrophil‐to‐CD8 ratio significantly decreased with the improvement of the pathology, the CD4‐to‐CD8 ratio remained stable at the different time points (Figure S5B). Our data suggest that although both CD4‐to‐CD8 and neutrophil‐to‐CD8 ratios are predictive of in‐ICU mortality of severe COVID‐19 cases ICU, the former appears to have stronger predictive potential as it remains stable throughout treatment in survivors.

## 4 DISCUSSION

In this study, we employed two flow cytometry panels to analyse whole‐blood samples from severe COVID‐19 patients admitted to ICU in order to describe the dynamics of myeloid and T cell subset populations during the critical phase of the disease.

Our analysis of the myeloid compartment showed comparable monocyte subset frequencies between survivors and non‐survivors. This is an important observation because although severe COVID‐19 cases share many of the clinical hallmarks of bacterial septic shock, these findings point towards key differences in the immune dysregulations underlying severe COVID‐19 to those we and others reported in sepsis.^21^, ^22^ In survivors, the relative frequency of classical monocytes increased significantly between ICU admission and restoration of spontaneous ventilation, accompanied by increased expression of HLA‐DR across all CD14+ mononuclear cells. Consistent with other studies, these results suggest the accumulation of dysfunctional and immunosuppressive monocytes with a myeloid‐derived suppressor cell‐like phenotype during the first phases of severe COVID‐19.^23^

When we compared the T cell subset composition in the blood of survivors and non‐survivors upon admission to ICU we observed lower counts of CM, EM and TEMRA subsets within total CD8+ T cells in subsequent non‐survivors, which was accompanied by an overall significantly lower frequency of total CD8+ T cells within the T cell compartment. Although reduced CD8+ T cell counts are known to be associated with the severity of this disease and eventual mortality, the difference in relative abundance of CD8+ T cell subsets might be due to distinct – and not exclusive – scenarios.^13^, ^20^ On one hand, a putative heightened inflammation would increase the migration of CM and EM T cells to the secondary lymph nodes and the inflamed lungs respectively.^24^ On the other hand, as it was recently demonstrated that acute COVID‐19 patients display higher proportions of CD8+ EM and TEMRA, the reduced counts observed in non‐survivors might underlie a defective activation of the CD8+ T cell arm.^15^ Finally, we can speculate that these disproportions in CD8 T cell subpopulations might also be already present in these patients even before the infection. Supporting this hypothesis, we did not observe any difference in the fraction of CD57+ cells among CD8+ TEMRA, suggesting that T cells might reach similar exhaustion levels irrespective of the outcome. Similarly, we failed to observe any differences between survivors and non‐survivors in the frequency of CD38+ HLA‐DR+CD8+ T cells, nor in the frequency of CCR5+ T cells, suggesting a similar degree of T cell activation. Moreover, we demonstrated that lower CD8+ CM, EM and TEMRA counts were associated with in‐ICU death. Interestingly, a recent study on a population of octogenarians demonstrated that higher counts of CD27‐ CD28+ CD8+ TEMRA protected against all‐cause death independently of CMV serostatus.^25^ Furthermore, it was also demonstrated that CD8+ CD57‐ TEMRA retain a functional, highly proliferative phenotype with differentiation plasticity.^26^ This suggests that the effector functions proposed already two decades ago, including Fas ligand expression, cytokine production and lysis efficacy of TEMRA (CD45RA+ CD27‐) mediated by T cell receptor, might be CD28‐dependent and decline with age.^27^, ^28^ Therefore, further investigations on CD8+ TEMRA are encouraged as the loss of CD28 expression might lead to impaired T cell functions and hence decreased in host protection during COVID‐19 and other viral infections.

Consistent with other recent studies we demonstrated that the neutrophil‐to‐CD8 ratio serves as a predictor of disease severity and in‐ICU mortality, and that the CD4‐to‐CD8 T cell ratio is a good prognostic marker for disease severity with mortality prediction power.^12^, ^20^, ^29^, ^30^, ^31^ However, the listed studies focusing predominantly on disease severity and outcome prediction did not link immunophenotypic markers with survivorship in in‐ICU patients specifically. As we found that in our cohort, the neutrophil‐to‐CD8 ratio decreased with disease recovery in survivors, we believe that this marker can be efficiently used to predict unfavourable outcomes and stratify the ICU population. Nevertheless, we did not observe a significant change in the CD4‐to‐CD8 ratio with recovery, suggesting that this parameter might be unbalanced independently of/prior to the disease and might, thus, identify a high‐risk population susceptible to lethal COVID‐19 complications. The CD4‐to‐CD8 ratio in the healthy population is very heterogeneous and is known to rise with age, with increased prevalence of high ratios (>5), especially after 50 years.^17^, ^18^, ^32^ Consistently, it was demonstrated that an increased CD4‐to‐CD8 ratio identifies a population of elders with impaired physical and mental performance, and lower reactivity against CMV antigens,^32^ and some studies have suggested that the CD4‐to‐CD8 T cell ratio is a risk factor for COVID‐19 critical illness and ICU admission.^29^, ^33^, ^34^

Our study indicates that while the neutrophil‐to‐CD8 ratio can be used as an early prognostic marker of COVID‐19 in‐ICU mortality, and, thus, to stratify the highest risk patients in ICU settings, the CD4‐to‐CD8 ratio might additionally be useful to identify a high‐risk population among currently‐healthy individuals. As well as representing a simple screening tool for older individuals, our study shows that this approach could also be valid for middle‐aged individuals: this is of particular importance as the 40‐49‐ and 50‐59‐year‐old groups currently account for more than 25% of cases requiring hospitalization for severe COVID‐19.^35^ As such, a larger prospective study to assess the relationship between high CD4‐to‐CD8 T cell ratio in otherwise‐healthy, or early‐stage/mild COVID‐19, in middle‐aged and older individuals and severe/fatal COVID‐19 is warranted.

Our study design has enabled us to effectively report the immune cell profiles of patients admitted to ICU with a very specific follow‐up time points regarding the actual health status of the patients – termination of ventilation support and their release from ICU. We were also able to track the mortality of patients with an exact time of their passing after ICU admission. This enabled us to assess the profiling not only within the survivors through their course of treatment but also differences in the immune cell profiles of survivor and non‐survivor groups. Nevertheless, there are limitations to this study. Due to the hospital personnel capacity and overall dynamic situation, we unfortunately lost track of few patients, mainly at the point of their release from ICU. Therefore, we were unable to perform proper paired statistical analyses. Moreover, fixation of the samples prior to their processing in our laboratory‐impeded cytokine analysis in the plasma and more detailed phenotyping regarding the antigen specificity to SARS‐CoV‐2. Despite these limitations, we have provided valuable insights with immediate clinical relevance to the treatment and monitoring of the sickest COVID‐19 patients and signposted directions for future research into risk stratification of the wider population for developing severe COVID‐19.

## CONFLICT OF INTEREST

The authors declare no competing interests.

## AUTHOR CONTRIBUTIONS

MDZ conceived the study, designed and performed experiments, analysed data, wrote the manuscript and secured funding; PL designed and performed experiments and wrote the manuscript; VT and MH recruited the patients and collected blood samples; MH and VŠ prepared the study protocol and the inclusion/exclusion criteria for patient enrolment; GF and GBS secured funding and provided advice; JF conceived and supervised the project, secured funding and oversaw the writing of the manuscript. All authors read and approved the final manuscript.

## ETHICAL APPROVAL

Written informed consent was obtained from the subjects after regaining consciousness. As for patients who failed to regain consciousness, anonymous data were processed with the consent of a relative. The study protocol complied with the Declaration of Helsinki and was approved by the institutional ethic committee (4G/2018).

## Supporting information

Supplementary Material

## Data Availability

The data that support the findings of this study are available from the corresponding author upon reasonable request.
