# Supplementary material for: High CD4‐to‐CD8 ratio identifies an at‐risk population susceptible to lethal COVID‐19
Source: Scand J Immunol. 2021 Dec 13;95(3):e13125. doi: 10.1111/sji.13125 (PMC9286348; doi:10.1111/sji.13125)
Supplement: Supplementary file 1 — Supplementary Material [file SJI-95-0-s001.docx]

**High CD4-to-CD8 ratio identifies an at-risk population susceptible to lethal COVID-19**

Marco De Zuani^1^, Petra Lazničková^1,2^, Veronika Tomašková^3^, Martina Dvončová^3^, Giancarlo Forte^1^, Gorazd Bernard Stokin^1,4^, Vladimir Šrámek^3^, Martin Helán^1,3^, Jan Frič^1,5,^*

^1^ International Clinical Research Center, St. Anne's University Hospital Brno, Brno, Czech Republic

^2^ Department of Biology, Faculty of Medicine, Masaryk University, Brno, Czech Republic

^3^ Department of Anaesthesiology and Intensive Care, Faculty of Medicine, Masaryk University, Brno, Czech Republic

^4^ Celica BIOMEDICAL, Tehnološki park 24, 1000, Ljubljana, Slovenia

^5^ Institute of Hematology and Blood Transfusion, Prague, Czech Republic

* Corresponding author: e-mail: [jan.fric@fnusa.cz](mailto:jan.fric@fnusa.cz), Pekarska 53, Brno 656 91, Czech Republic

**SUPPLEMENTARY INFORMATION**

**Supplementary Figure 1**

**
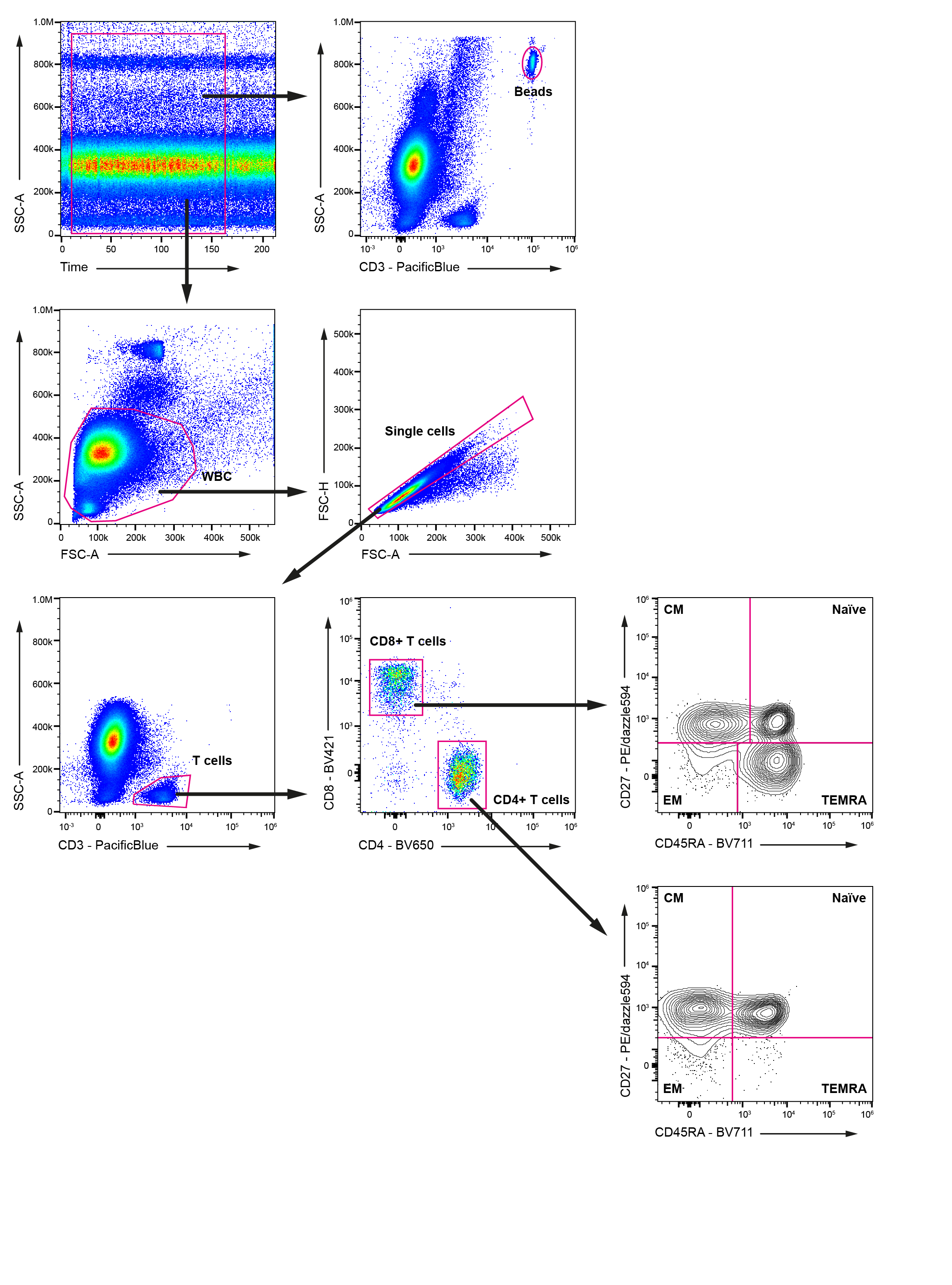
**

**Supplementary Figure 1: Gating strategy used for the T cell flow cytometry panel.** An initial time gate was set to exclude regions with poor flow rate. Beads were then detected as SSC^hi^, PacificBlue^bright^. Total white blood cells (WBC) were identified on their scatter morphology, and single cells were gated using FSC-A vs FSC-H. T cells were then identified as SSC^lo^, CD3+ and further sub-gated into CD8+ and CD4+ populations. Both T cell subsets were further characterised as naïve (CD45RA+, CD27+), CM (CD45RA-, CD27+), EM (CD45RA-, CD27-) and TEMRA (CD45RA+, CD27-).

**Supplementary Figure 2**

**
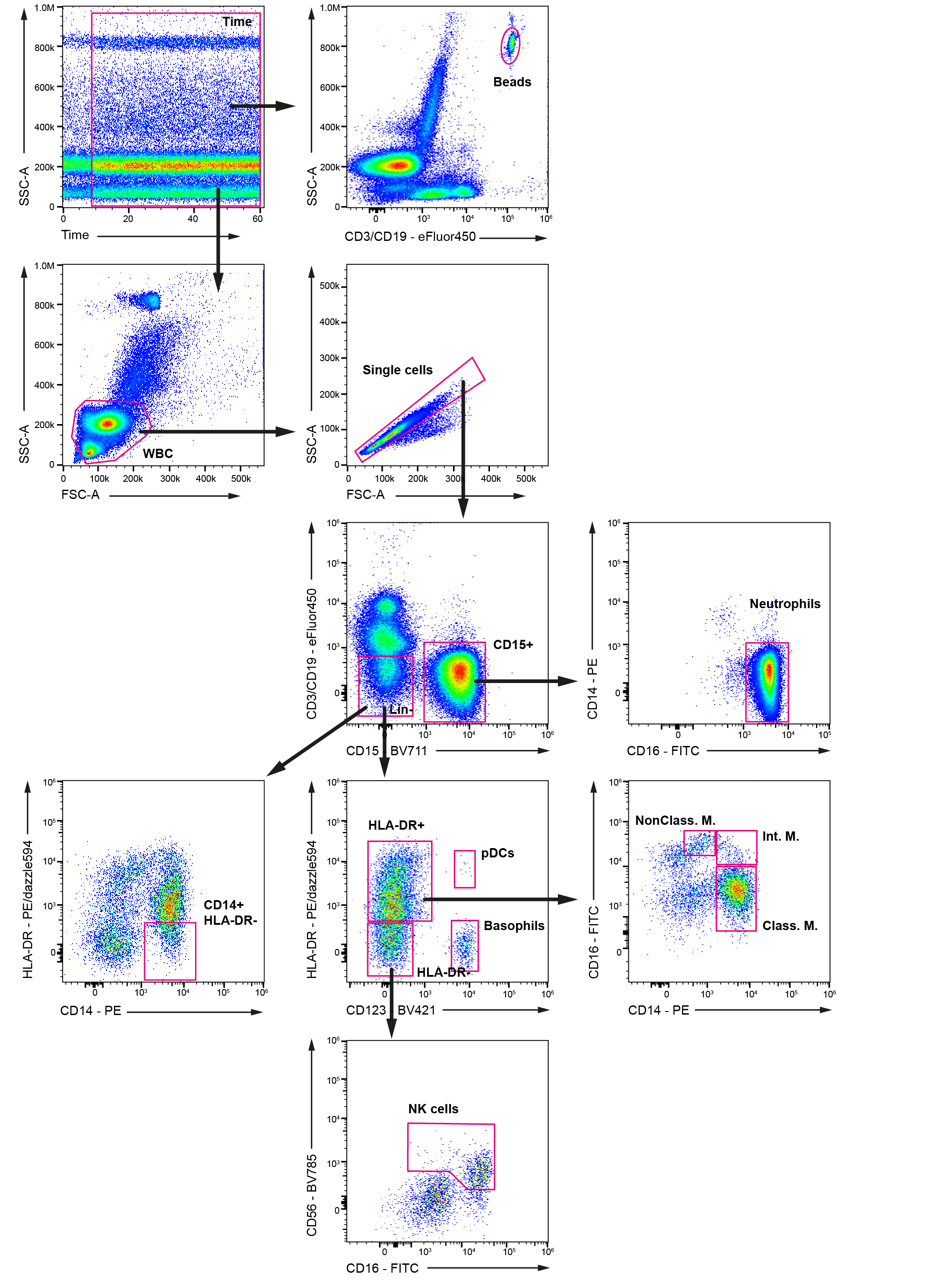
**

**Supplementary Figure 2: Gating strategy used for the myeloid cell flow cytometry panel.** An initial time gate was set to exclude regions with poor flow rate. Beads were then detected as SSChi, eFluor450bright. Total white blood cells (WBC) were identified on their scatter morphology, and single cells were gated using FSC-A vs FSC-H. CD15+, Lin(CD3/19)- cells were further sub-gated to identify neutrophils (CD15+, CD16+, CD14-). CD15-, Lin(CD3/19)- cells were used to identify CD14+ HLA-DR- monocytes. Similarly, plasmacytoid dendritic cells - pDCs (HLA-DR+, CD123+) and basophils (HLA-DR-, CD123+) were identified among the Lin- population. HLA-DR+ cells were further sub-gated to identify the main monocyte populations: classical monocytes (CD14hi, CD16-), intermediate monocytes (CD14hi, CD16+) and non-classical monocytes (CD14lo, CD16+). Finally, natural killer cells (NKs) were identified among the HLA-DR- population as CD56+/dim, CD16+/-.

**Supplementary Figure 3**

**
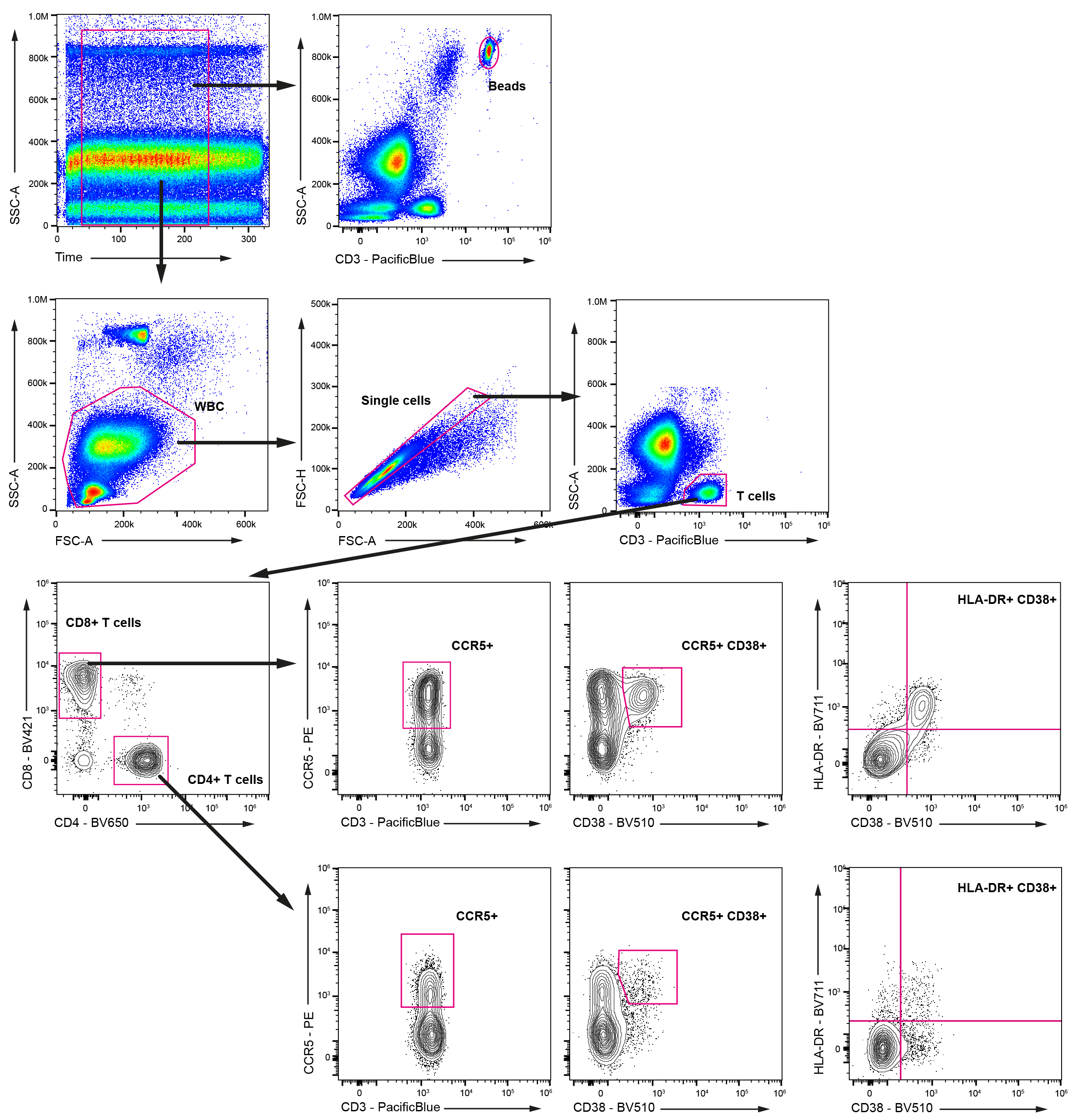
**

**Gating strategy used for the identification of activated T cells.** An initial time gate was set to exclude regions with poor flow rate. Beads were then detected as SSC^hi^, PacificBlue^bright^. Total white blood cells (WBC) were identified on their scatter morphology, and single cells were gated using FSC-A vs FSC-H. T cells were then identified as SSC^lo^, CD3+ and further sub-gated into CD8+ and CD4+ populations. Both T cell subsets were further characterised as on the basis of their expression of HLA-DR, CD38 and CCR5.

**Supplementary Figure 4**

**
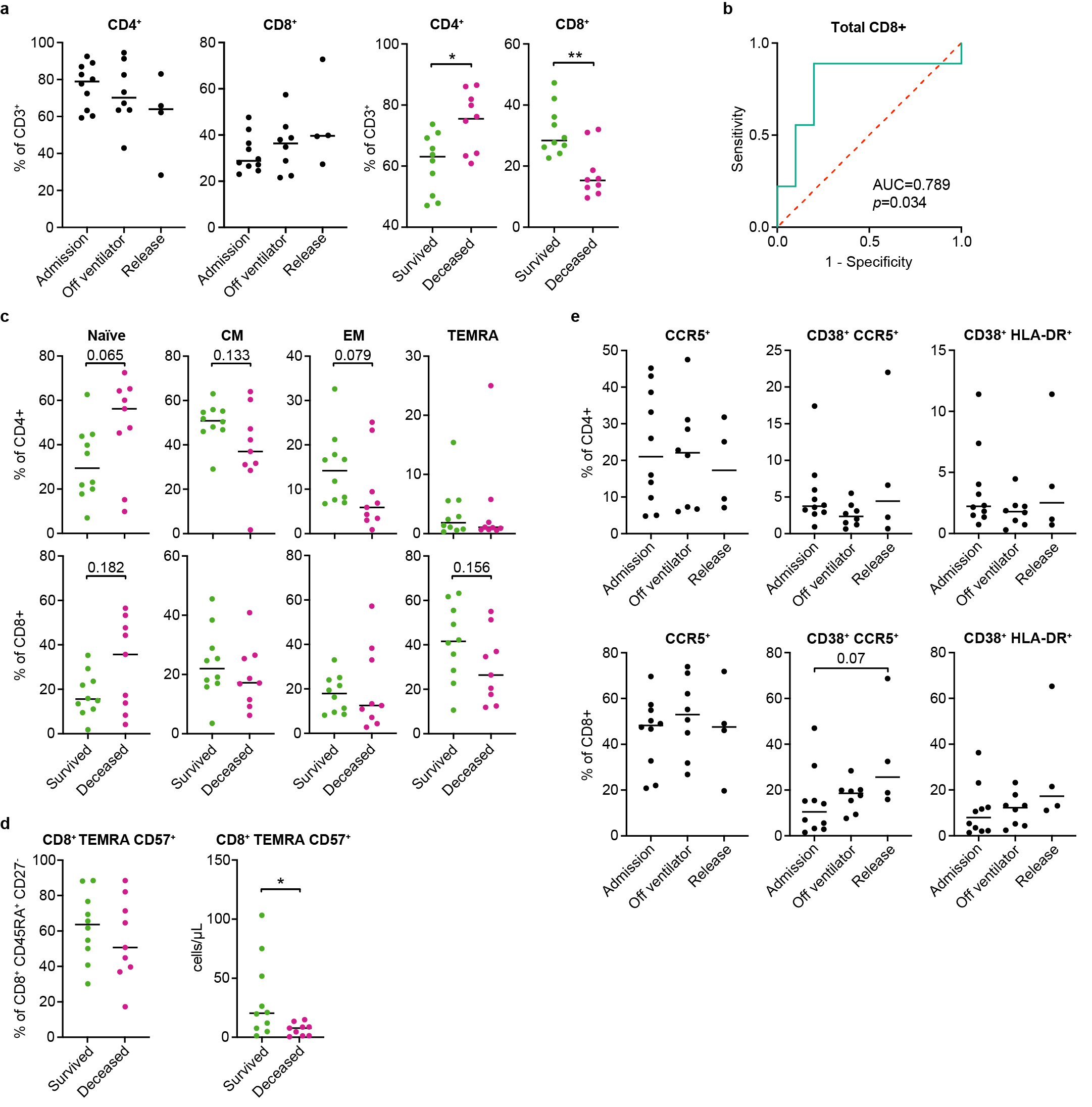
Supplementary Figure 4: T cell subset counts. a** Frequencies of CD4+ and CD8+ T cell subsets measured at different time points in severe COVID-19 survivors (left), and compared at ICU admission between severe COVID-19 survivors and non-survivors (right). **b** ROC curve for total CD8+ T cells. **c** Frequencies of naïve, CM, EM and TEMRA subsets in both CD4+ and CD8+ T cell subsets, measured at ICU admission and compared between survivors and non-survivors. **d** Frequencies of CD57+ CD8+ TEMRA (left) and total CD57+ CD8+ TEMRA counts in survivors and non-survivors. **e** Frequencies of CD38+ HLA-DR+, CD38+ CCR5+, and CCR5+ T cells in both CD4+ (top) and CD8+ (bottom) subsets measured at different time points in severe COVID-19 survivors. Multiple comparisons in panel **a** and **e** were performed with a Kruskal-Wallis test with Dunn’s correction. Single comparisons in panels **a,** **c** and **d** were performed with a Mann-Whitney test. Asterisk indicates the level of significance. * *p* < 0.05, ** *p* < 0.01.

**Supplementary Figure 5**


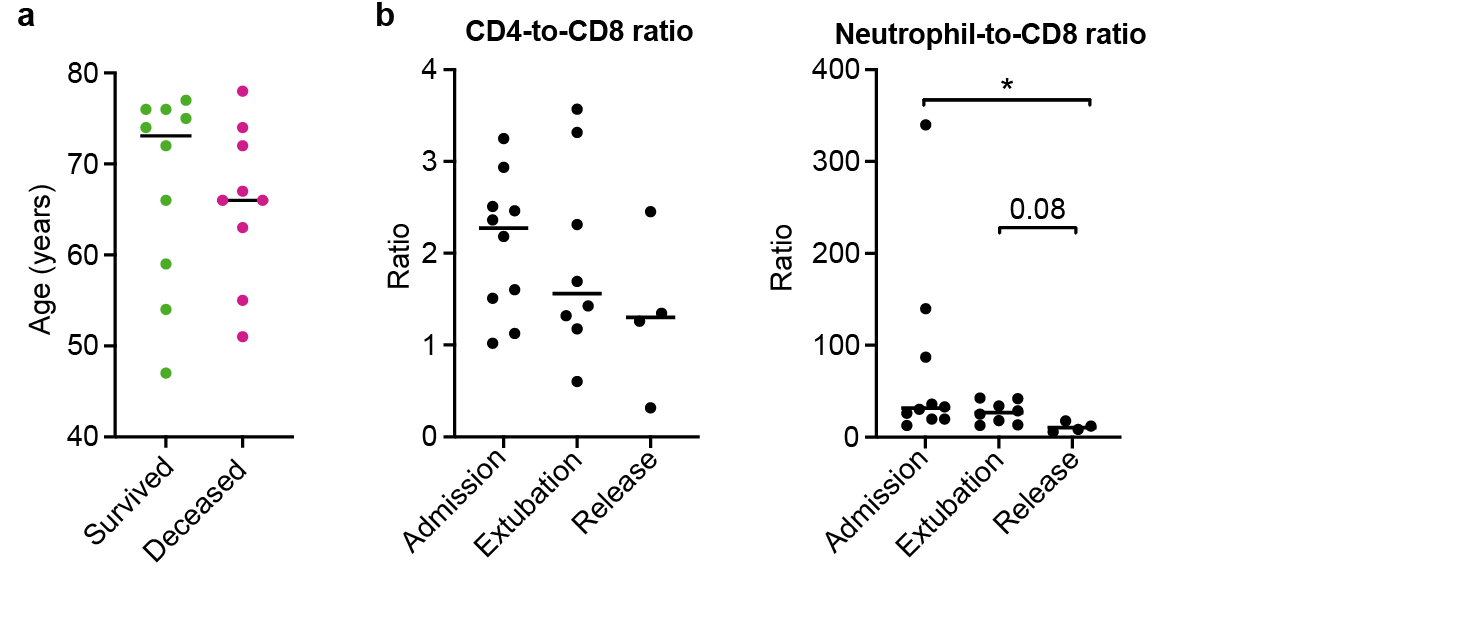


**Supplementary Figure 5: Temporal changes in CD4-to-CD8 T cell ratio and neutrophil-to-CD8 T cell ratio.** **a** Age distribution in severe COVID-19 survivor and non-survivor groups. **b** CD4-to-CD8 ratio and neutrophil-to-CD8 ratio measured at different time points in severe COVID-19 survivors. Data in **a** were analysed by Mann-Whitney test. Data in **b** were analysed by Kruskal-Wallis test with Dunn’s correction. Asterisk indicates the level of significance. * *p* < 0.05.

**Supplementary Table 1**

**Panel 1 – T cell subsets**

| **Marker** | **Conjugation** | **Dilution** | **Clone** | **Vendor** | **Cat. #** | **Lot #** |
| --- | --- | --- | --- | --- | --- | --- |
| CD3 | PB | 1:100 | OKT3 | BioLegend | 317314 | B276555 |
| CD4 | BV650 | 1:100 | SK3 | BD Horizon | 563875 | 9107661 |
| CD8 | BV421 | 1:50 | RPA-T8 | BioLegend | 301036 | B3020687 |
| CD45RA | BV711 | 1:100 | HI100 | Sony | 2120690 | 150703 |
| CD45RO | AF488 | 1:100 | UCHL1 | BioLegend | 304212 | B240517 |
| CD27 | PE/dazzle594 | 1:200 | M-T271 | Sony | 2382110 | 191375 |
| CD57 | PE/Cy7 | 1:50 | HNK-1 | BioLegend | 359624 | B268749 |

**Panel 2 – Myeloid subsets**

| **Marker** | **Conjugation** | **Dilution** | **Clone** | **Vendor** | **Cat. #** | **Lot #** |
| --- | --- | --- | --- | --- | --- | --- |
| CD3 | Biotin | 1:100 | UCHT1 | Invitrogen | 13-0038-82 | 4341639 |
| CD19 | Biotin | 1:100 | HIB19 | Sony | 2111020 | 113895 |
| Streptavidin | eF450 | 1:100 | - | Invitrogen | 48-4317-82 | 1988686 |
| CD14 | PE | 1:50 | 61D3 | Invitrogen | 12-0149-42 | 2022015 |
| CD16 | FITC | 1:100 | 3G8 | BioLegend | 302006 | B256873 |
| CD15 | BV711 | 1:100 | W6D3 | Sony | 2215250 | 165326 |
| CD56 | BV785 | 1:50 | 5-1H11 | BioLegend | 362550 | B303958 |
| HLA-DR | PE/dazzle594 | 1:25 | L243 | BioLegend | 307654 | B246984 |
| CD123 | BV421 | 1:100 | 6H6 | Sony | 2130090 | 153023 |

**Panel 3 – T cell activation**

| **Marker** | **Conjugation** | **Dilution** | **Clone** | **Vendor** | **Cat. #** | **Lot #** |
| --- | --- | --- | --- | --- | --- | --- |
| CD3 | PB | 1:100 | OKT3 | BioLegend | 317314 | B276555 |
| CD4 | BV650 | 1:100 | SK3 | BD Horizon | 563875 | 9107661 |
| CD8 | BV421 | 1:50 | RPA-T8 | BioLegend | 301036 | B3020687 |
| HLA-DR | BV711 | 1:100 | L243 | BioLegend | 307644 | B305688 |
| CD38 | BV510 | 1:100 | HB-7 | Sony | 2383060 | 201711 |
| CCR5 (CD195) | PE/dazzle594 | 1:200 | J418F1 | BioLegend | 359126 | B222944 |

**Supplementary Table 2**

|  | **Age** | **BMI** | **CD4-to-CD8** | **Neut.-to-CD8** | **CD8 count** | **CD4 count** | **T cell count** | **Comorbidities** | **SOFA** | **CRP** | **Horowitz** | **Leukocytes** | **Creatinine** |
| --- | --- | --- | --- | --- | --- | --- | --- | --- | --- | --- | --- | --- | --- |
| **Pearson r** | -0.09776 | 0.1875 | 0.6738 | 0.2806 | -0.3037 | -0.05426 | -0.1425 | 0.3271 | 0.2823 | -0.1084 | 0.0296 | -0.2527 | 0.3083 |
| **95% C.I.** | -0.5285 to 0.3730 | -0.2916 to 0.5913 | 0.3165 to 0.8637 | -0.1989 to 0.6518 | -0.6660 to 0.1746 | -0.4962 to 0.4101 | -0.5605 to 0.3333 | -0.1493 to 0.6803 | -0.1972 to 0.6528 | -0.5599 to 0.3927 | -0.4304 to 0.4774 | -0.6436 to 0.2428 | -0.2023 to 0.6871 |
| **R^2^** | 0.009558 | 0.03515 | 0.4541 | 0.07875 | 0.09224 | 0.002944 | 0.02031 | 0.107 | 0.07967 | 0.01175 | 0.0008763 | 0.06387 | 0.09504 |
| ***p*** | 0.6905 | 0.4421 | 0.0016 | 0.2445 | 0.2062 | 0.8254 | 0.5605 | 0.1716 | 0.2417 | 0.6788 | 0.9042 | 0.3116 | 0.2286 |

**Supplementary Table 2: Correlation between mortality and clinical and measured parameters.** Correlations in were calculated by a Pearson correlation test. SOFA: sequential organ failure assessment; CRP: C-reactive protein, Horowitz index (PaO_2_/FiO_2_): lung function index defined as the ratio of partial pressure of oxygen in arterial blood (PaO_2_) to the inspiratory fraction of oxygen (FiO_2_) .
